# Effects of Intra-Articular Stromal Vascular Fraction Injection on Clinical Symptoms and Cartilage Health in Osteoarthritic Knees: A Single-Center Pilot Study

**DOI:** 10.3390/life14111468

**Published:** 2024-11-12

**Authors:** Chun-Ru Lin, Chia-Nan Lin, Chung-Chen Lee, Yong-Chen Chen, Yu-Jen Chen, Ming-Hao Chen, Yu-Chih Lin, Shu-Hao Chang

**Affiliations:** 1 Department of Orthopedic Surgery, Chang Gung Memorial Hospital, Linkou Branch, No. 5, Fuxing Street, Guishan District, Taoyuan City 33305, Taiwan; asd450132@gmail.com (C.-R.L.); b101092127@cgmh.org.tw (Y.-C.L.); 2 Department of Medical Imaging, Fu Jen Catholic University Hospital, Fu Jen Catholic University, New Taipei City 24352, Taiwan; a00411@mail.fjuh.fju.edu.tw; 3 Data Science Center, College of Medicine, Fu Jen Catholic University, No. 510, Zhongzheng Rd., Xinzhuang District, New Taipei City 24205, Taiwan; rainbow2748@gmail.com (C.-C.L.); yongchenchen0824@gmail.com (Y.-C.C.); 4 School of Medicine, College of Medicine, Fu Jen Catholic University, No. 510, Zhongzheng Rd., Xinzhuang District, New Taipei City 24205, Taiwan; 408510095fjumed@gmail.com; 5 Research and Development Center for Physical Education, Health, and Information Technology, Fu Jen Catholic University, New Taipei City 24205, Taiwan; yujenc@gmail.com; 6 Department of Orthopedics, Fu Jen Catholic University Hospital, Fu Jen Catholic University, No. 69, Guizi Rd., Taishan District, New Taipei City 24352, Taiwan

**Keywords:** osteoarthritis, intra-articular stromal vascular fraction injections, stromal vascular fraction, MRI T2 mapping, clinical questionnaires, stem cell therapy

## Abstract

Osteoarthritis (OA) is a prevalent form of arthritis worldwide. Intra-articular stromal vascular fraction (SVF) injections are a potential therapeutic option for patients with OA. This study aims to assess the effects of intra-articular SVF injections on knee OA. Ten patients with knee OA participated in this study. After administering them with intra-articular SVF injections, their outcomes were evaluated using various questionnaires. MRI T2 mapping was conducted and compared before the intervention and 6 months after. All the data underwent analysis using various tests. Significant differences were observed in the change of Western Ontario and McMaster Universities Arthritis Index, VAS, and Knee Injury and Osteoarthritis Outcome Score between pre-intervention and 6 months post-intervention. The T2 values were significantly lower in the anterior superficial layer of the medial femoral cartilage and middle superficial layer of the lateral femoral cartilage. However, no positive effects were observed in any other regions of the knee cartilage. This study revealed significant differences between the pre- and 6-month post-intervention questionnaires. However, the T2 values did not show consistent changes across all regions of the knee cartilage. Despite positive effects in two regions, the degenerative process appeared to continue in other regions during the tracking period.

## 1. Introduction

Osteoarthritis (OA) is a debilitating disease and is endemic globally. Annually, approximately 30.8 million individuals in the USA and 300 million individuals worldwide are affected by OA [1], which is especially prevalent in aging populations and can significantly limit mobility and quality of life [2]. Among men aged 60 to 64, the right knee tends to be more frequently affected, while in women, both knees are almost equally impacted [2]. In terms of the incidence of knee OA, the prevalence is higher in females compared to males [3]. Furthermore, among individuals aged 50 and above, the risk of developing knee OA in females is approximately twice that of males [4]. In Taiwan, approximately 37% of adults over the age of 50 have OA [5]. The annual medical costs associated with OA in the USA are estimated to be USD 303 billion [1]. The most common symptoms of OA are pain and loss of motion and function of the affected joint [6]. There is often a mismatch between the intensity of symptoms and the radiographic severity of knee osteoarthritis. This inconsistency may be attributed to factors such as pain sensitization, adaptation to chronic pain, or a reduction in physical activity to mitigate discomfort [7]. For ACL problems, a combination of lever signs, pivot shifts, and Lachman tests demonstrated better sensitivity and specificity in detecting ACL deficiencies compared to MRI [8]. Treatments for OA include medication, nonpharmacological and alternative measures, and surgery [9]. Its poor self-repair capacity and lack of specific biomarkers make it difficult to treat, with current therapies like acetaminophen and NSAIDs offering only pain relief and often causing side effects. Newer drugs, such as sprifermin and tanezumab, are being developed to improve effectiveness, with fewer side effects. Additionally, regenerative therapies like autologous chondrocyte implantation (ACI) and induced pluripotent stem cells (iPSCs) show potential for enhancing cartilage repair. Despite advancements, unmet medical needs persist in OA treatment [10]. These treatments primarily delay the progression of symptoms rather than prevent the loss of cartilage [11].

Currently, stem cell therapies such as mesenchymal stem cells (MSCs) and adipose-derived stem cells (ASCs) are potential disease-modifying treatments for OA owing to their multipotent properties for differentiation into chondrocytes [12]. Nevertheless, isolating MSCs and ASCs in laboratories requires several weeks [13]. A more efficient measure to obtain ASCs is through stromal vascular fraction (SVF) cells. SVF comprises heterogeneous cells surrounding the adipose tissue, including stromal cells, ASCs, vascular endothelial cells, and their progenitor cells. SVF cells can differentiate into various cell types, including chondrocytes, osteoblasts, myocytes, hepatocytes, and hematopoietic and neural cells [14]. Adipose tissues can easily be obtained via liposuction harvest to isolate SVF cells subsequently. The SVF process does not require cell culture, and the entire process can be completed at the bedside or in the operating room. A previous double-blinded, prospective, randomized controlled clinical trial in the USA indicates that intra-articular SVF injections can decrease the symptoms of OA for 12 months [11]. Therefore, this study aims to quantitatively assess the effects of intra-articular SVF injections on patients with knee OA in Asia using clinical questionnaires and quantitative MRI T2 mapping.

The primary aim of this study is to quantitatively assess the therapeutic effects of intra-articular SVF injections on knee OA in an Asian population. By utilizing MRI T2 mapping, a sensitive and non-invasive imaging technique, we aim to evaluate changes in cartilage composition and health following treatment. This study combines clinical assessments and advanced imaging to provide a comprehensive understanding of SVF’s potential to repair cartilage and improve joint function.

## 2. Materials and Methods

### 2.1. Study Overview

This study was conducted between July 2021 and June 2022 as a single-center, prospective clinical trial with a standard 6-month follow-up period at the Fu Jen Catholic University Hospital, New Taipei City, Taiwan. The study was approved by the Fu Jen Catholic University Institutional Review Board (No. FJUH109069). All patients included in this study provided written informed consent before the commencement of the trial.

### 2.2. Patient Population

This prospective clinical trial included 10 patients based on the following inclusion and exclusion criteria.

Inclusion criteria included the following:

1. Aged ≥ 35 years.
2. Patients with degenerative knee OA confirmed via X-ray and classified according to the Kellgren Lawrence Grading Scale 2~4.
3. Persistent knee pain after nonsurgical treatment.

Exclusion criteria:

1. Arthritis caused by other systemic or local immune diseases.
2. Patients who had undergone total knee replacement surgery.
3. Patients who had been administered platelet-rich plasma or hyaluronic acid injections within the past three months.
4. Patients with symptoms related to degenerative arthritis in the ankle, which affected the assessment of pain.
5. Patients with severe heart problems and coagulation disorders.
6. Patients who had taken glucosamine within the previous month.
7. MRI exclusion conditions, including patients with a heart rhythm regulator who had received vascular surgery with a hemostatic clip and artificial heart valves.

### 2.3. Intervention

The preparation process of SVF follows established methodologies from previous research [15]. Adipose tissue was harvested using the Q-graft system (Human Med^®^, Schwerin, Germany) from the abdominal subcutaneous fat of patients under local anesthesia. An average of 75 mL of adipose was directly collected and concentrated in the Q-graft collector, with waste fluid removed via the body-jet system. The lipoaspirate was mixed and incubated at 38 °C for 45 min, and then an average of 20 mL of SVF cells was isolated.

After concentration and resuspension in normal saline, 5 mL of the SVF suspension was injected into the knee joint cavity under sterile conditions. The injection was per-formed over 1–2 min at a constant speed. After the injection of SVF suspension, the patients were observed for 30 min. If no adverse local reactions occurred, they were allowed to leave the hospital.

### 2.4. Outcome Measures

Clinical outcomes were assessed prior to the intervention and at 3 and 6 months post-intervention. The outcomes included the Western Ontario and McMaster Universities Osteoarthritis Index (WOMAC), which ranges from 0 to 96, with higher scores indicating greater osteoarthritis-related disability [16]; the Knee Injury and Osteoarthritis Outcome Score (KOOS), consisting of 5 items and also ranging from 0 to 96, where higher scores reflect a poorer quality of life related to osteoarthritis [17]; and the Visual Analogue Scale (VAS) [18], which ranges from 0 to 10, with higher scores signifying an increased level of pain. Magnetic resonance imaging (MRI) included T2 mapping conducted both prior to the intervention and 6 months after the intervention.

### 2.5. Image Acquisition

All MRI scans were conducted using a 3-T MRI scanner (Ingenia 3T, Philips Health System, Best, The Netherlands) with a 16-channel transmit–receive phased-array knee coil. No specific activity protocol was employed before MR imaging. All patients were able to perform routine daily activities and walk to the MR imaging suite independently. During scanning, the patient lay supine with both knees fully extended and relaxed. All scans were conducted in the same session. T2 mapping was performed employing a sagittal 2D multi-echo spin-echo sequence with 6 echoes using the following parameters: TR, 2000 ms; TEs, 13, 26, 39, 52, 65, and 78 ms; 90° flip angle; field of view, 160 × 160 mm; matrix, 296 × 236 (interpolated to 256 × 256); bandwidth, 290 Hz per pixel; voxel size, 0.54 × 0.68 × 3 mm; and slice thickness 3 mm, gap 0.3 mm. The acquisition time was 5 mi 36 s. T2 relaxation times were calculated from online-reconstructed T2 maps using pixelwise, monoexponential, nonnegative least-squares fit analysis (IntelliSpace Portal v10.1, Philips, The Netherlands). Other pulse sequences for morphological evaluation included a T1-weighted sequence in the coronal plane (FOV 140 × 140 × 100 mm, TR/TE/NEX 663/20/1, matrix 280 × 231 × 40, voxel size, 0.5 × 0.6 × 2.6 mm, slice thickness 2.5 mm, and gap 0 mm), and proton-density-weighted sequence with fat-saturated sequence in the axial plane (FOV 170 × 170 mm, TR/TE/NEX 4925/30/1, matrix 339 × 270, slice thickness 2.5 mm, and gap 0 mm). Table 1 details the setup, allowing for comprehensive morphological evaluation.

### 2.6. Image Evaluation

**Figure 1:**
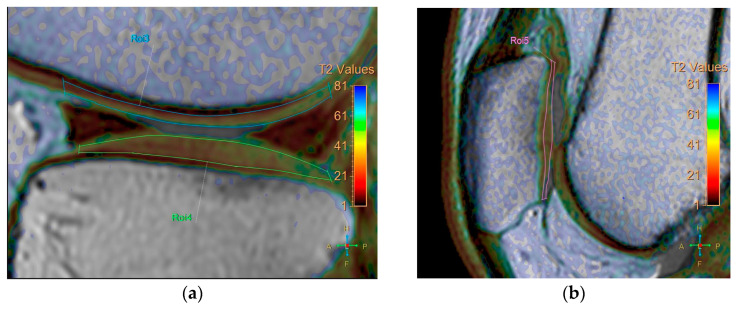
Zonal region of interest (ROI) analysis was performed in sagittal view in the main weight-bearing region, defined based on the peripheral margins of the menisci for the femorotibial joint and from the areas with the largest sagittal cross-sectional dimension of the patellofemoral joint. (**a**) coronal view. (**b**) sagittal view.

**Figure 2:**
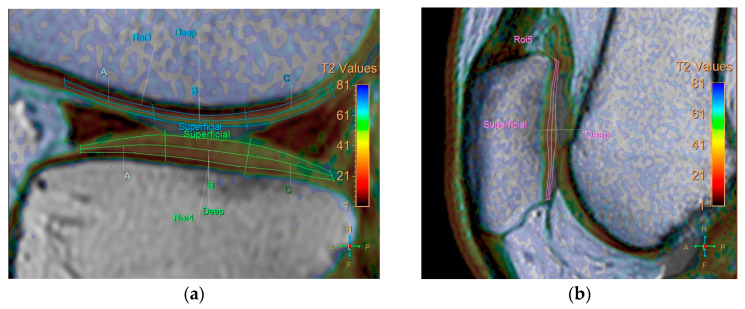
Division of ROIs into deep and superficial layers. The femoral and tibial cartilages were further divided into anterior, middle, and posterior segments. (**a**) coronal view. (**b**) sagittal view.

All baseline and follow-up MR images were anonymized and randomized before analysis by one orthopedic surgeon, one radiologist (with 8 and 7 years of experience, respectively), and a resident, all of whom were blinded to the identities of the patients and the timing of the examinations. Five regions were defined for cartilage evaluation: the medial and lateral femoral condyle, medial and lateral tibia plateau, and patella. Cartilage thickness and T2 measurements were obtained in these regions. To verify the localization of the cartilage, we used high-spatial-resolution, morphologic, isotropic, three-dimensional multiple fast field echo imaging in sagittal view. Imaging parameters included the following: field of view, 140 × 140 × 100 mm; matrix, 176 × 174; voxel size, 0.8 × 0.8 × 1 mm; and slice thickness, 1 mm, gap −0.5 mm. T2-weighted images and maps were assessed side by side using a Philips workstation (IntelliSpace Portal (ISP, Philips Health System) v10.1, Best, The Netherlands). We manually outlined the articular cartilage, excluding any subchondral bone and synovial fluid. Zonal ROI analysis was conducted in sagittal view in the main weight-bearing region, which was defined based on the peripheral margins of the menisci for the femorotibial joint and the areas with the largest sagittal cross-sectional dimension of the patellofemoral joint (Figure 1). All ROIs covered the calculated full thickness of cartilage. For further evaluation of the zonal variation in T2 measurements, the ROIs were divided into deep and superficial layers based on the thickness of the cartilage, and into anterior, middle, and posterior segments (Figure 2).

Cartilage thickness was measured based on a vector perpendicular to the surface of the subchondral femur, tibia, and patella at the center of the three segments. The analysis took 40 min per patient. Each ROI was measured twice, with a one-month interval between measurements, to obtain average T2 values to mitigate potential errors.

### 2.7. Statistical Analysis

To describe the characteristics of the sample, we assessed the normal distribution of the data using the Shapiro–Wilk test. Normally distributed data were represented as mean values ± standard deviation, while non-normally distributed data were presented as a median ± interquartile range. In addition, the continuous variables were analyzed using paired *t*-tests (normal distribution) and Wilcoxon signed-rank test (non-normal distribution). Inter-observer and intra-observer agreements were assessed using the intraclass correlation coefficient. Spearman’s rank correlation coefficients were calculated to determine the correlations between relative changes in clinical questionnaires and T2 values of the cartilage. In this study, SAS 9.4 statistical software was used, and the significance level (α) was set to 0.05. Two-tailed tests were used for data analysis.

## 3. Results

Initially, 10 participants were enrolled in the trial. However, one case was excluded due to the participant’s inability to attend regular follow-up appointments because of work commitments, resulting in the absence of post-treatment functional scores and MRI data. Consequently, a total of nine participants completed the trial. The final cohort of nine participants included solely female subjects. Table 2 shows the characteristics of the patients in our trial. The mean age was 58.22 years, with a standard deviation (SD) of 5.29, and with a median age of 59 years and an interquartile range (IQR) of 7 years. The patients ranged in age between 50 and 69 years. The 50–54-, 55–59-, 60–64-, and 65–69-year-old groups accounted for proportions of the overall total of patients at 22.22%, 33.33%, 33.33%, and 11.11%, respectively. Nine patients were assessed for knee OA, with five (55.55%) classified as Grade 2 and three (33.33%) as Grade 3 in the right knee; one patient (11.11%) had undergone knee replacement. In the left knee, seven patients (77.77%) were Grade 2 and two patients (22.22%) were Grade 3. In addition, the median total live cell count of SVF was 2,810,000, with an interquartile range (IQR) of 1,140,000.

Table 3 shows the scores of the questionnaires at different times. The Western Ontario and McMaster Universities Arthritis Index (WOMAC) scores were 41.00 ± 25.67 (means ± SD) pre-intervention, 16.33 ± 13.04 at 3 months post-intervention, and 20.22 ± 19.01 at 6 months after the intervention. The Visual Analogue Scale (VAS) scores were 5.00 ± 2.96 pre-intervention, 3.89 ± 3.76 3 months after the intervention, and 3.00 ± 3.50 6 months post-intervention. The adjusted Knee Injury and Osteoarthritis Outcome Score (KOOS) scores were 52.01 ± 14.67 pre-intervention, 61.96 ± 13.41 at 3 months post-intervention, and 63.02 ± 16.85 at 6 months after the intervention.

Table 4 compares the scores of the questionnaires at different times. The WOMAC scores showed significant improvement between pre-intervention and 3 months after the intervention (24.67 ± 25.00, *p* = 0.0181), as well as between pre-intervention and 6 months post-intervention (20.78 ± 7.03, *p* = 0.0183). The VAS scores showed significant improvement between pre-intervention and 6 months after the intervention (2.00 ± 2.29, *p* = 0.0307). The KOOS scores showed significant improvement between pre-intervention and 6 months post-intervention (−11.01 ± 13.93, *p* = 0.0453).

For the T2 values of the cartilage measurement, the inter-observer agreement among the three observers was 0.600, and the intra-observer agreement values were 0.594, 0.505, and 0.608.

The baseline T2 values for cartilage before SVF treatment are presented in median ± IQR. Overall, the values were 51.1 ± 14.3 ms for the medial femoral condyle, 47.9 ± 13.1 ms for the lateral femoral condyle, 40.8 ± 9.6 ms for the medial tibial plateau, and 30.9 ± 4.5 ms for the lateral tibial plateau (Table 5). The T2 values for the medial femorotibial joint were significantly higher than those for the lateral femorotibial joint (*p* = 0.0002). Furthermore, the T2 values for the femoral condyles were significantly higher than those for the tibial plateaus (*p* < 0.0001). The T2 values for the patella cartilage were 45.9 ± 10.1 ms. The zonal region of interest (ROI) was divided into deep and superficial layers, based on the thickness of the cartilage (Table 6). In all joint compartments, the T2 values for the superficial layers were significantly higher than those of the deep layers (*p* < 0.0001).

Table 7 lists all the T2 values for cartilage in each segment. The T2 values were significantly lower than those in the baseline after SVF treatment in the anterior superficial layer of medial femoral cartilage, with relative changes of 6.7 ± 11.2 ms (*p* = 0.0052), and the middle superficial layer of lateral femoral cartilage, with relative changes of 3.7 ± 4.4 ms (*p* = 0.0131). However, the T2 values showed a significant increase in the posterior superficial layer of the medial tibial cartilage, with relative changes of −2.8 ± 3.5 ms (*p* = 0.0027). In most of the regions of cartilage in the tibia, femur, and patella, no significant differences were observed in T2 values before and after SVF treatment.

**Figure 3:**
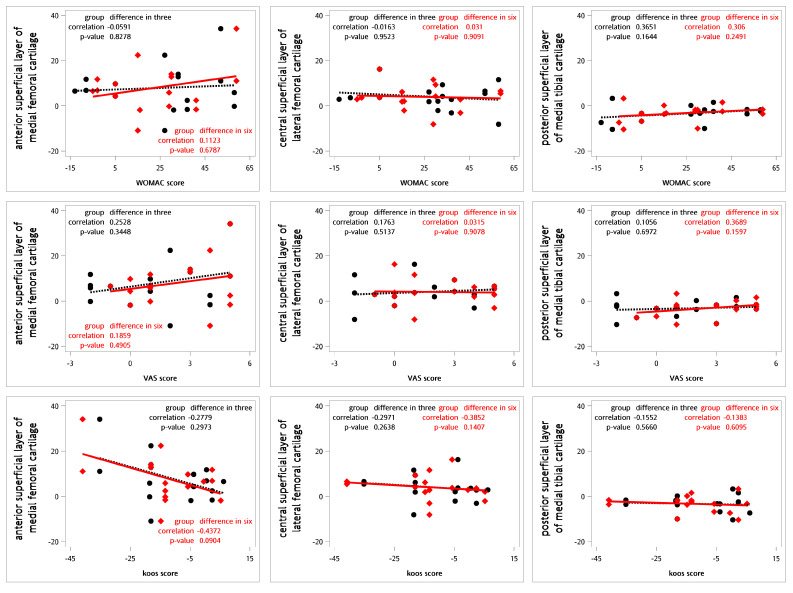
Correlations between the relative differences in clinical questionnaires and T2 values using Spearman’s correlation coefficient (n = 9). The black dot represents the change in T2 values and clinical questionnaire scores three months following intra-articular SVF injections. The red diamond indicates the change in T2 values and clinical questionnaire scores six months after the same treatment. The black dashed line illustrates the linear regression of the black dots, while the red line depicts the linear regression of the red diamond points.

For testing the correlations between the relative changes in the questionnaires and the T2 values for cartilage, Spearman’s rank correlation coefficients were calculated (Figure 3). No definite correlations were observed between the relative changes in T2 values and the WOMAC, VAS, and KOOS scores at 3 or 6 months (Table 8).

Table 9 lists all the cartilage thicknesses in every segment. In most of the regions of cartilage in the tibia, femur, and patella, no significant differences were observed in the cartilage thickness before and after SVF treatment. Only the middle segment in the lateral femur showed significantly thinner changes after SVF, with relative changes of 0.1 ± 0.2 mm (*p* = 0.0131).

## 4. Discussion

In our study, significant differences were observed in the changes of WOMAC (20.78 ± 7.03, *p* = 0.0183), VAS (2.00 ± 2.29, *p* = 0.0307), and KOOS (−11.01 ±13.93, *p* = 0.0453) be-tween pre-intervention and 6 months post-intervention. In addition, SVF demonstrated a positive effect on the anterior superficial layer of the medial femoral cartilage and middle superficial layer of the lateral femoral cartilage, while it had the opposite effect on the posterior superficial layer of the medial tibial cartilage.

OA is the most common chronic joint disease [19], and its occurrence increases with age. In the United States, approximately 37.4% of individuals aged > 60 are affected by OA [20]. In the early stages of OA, the surface of the cartilage remains intact while the components of the extracellular matrix change [21]. This consequently triggers a short-term proliferation of chondrocytes in the healthy joints, leading to increased synthesis of matrix components and hypertrophy of chondrocytes [21]. The related hypertrophic markers include Colx, Runx2, and Mmp 13 [21]. The changes mentioned above stimulate chondrocytes to synthesize the metabolic factors that degrade the chondrocytes, resulting in collagen degradation, loss of collagen integrity, and subsequent apoptosis of chondrocytes [21]. Microscopically, OA cartilage shows a loss of collagen and proteoglycans [22], disrupting the extracellular matrix and impairing biomechanical properties [23]. Chondrocytes in the superficial layer form clusters, while those in deeper layers undergo apoptosis [10]. Although chondrocyte proliferation is somewhat increased, it cannot overcome the prevailing catabolic activity [24]. OA chondrocytes produce matrix-degrading enzymes, such as MMP13 and ADAMTS-5, which further accelerate cartilage breakdown [25]. Owing to the narrowing joint space, the friction within the joints increases, which results in pain, less range of motion, and poor quality of life [21]. Owing to the limited understanding of the underlying pathogenesis and mechanism of OA, efficient interventions to prevent cartilage from degenerating remain elusive [26].

Due to the side effects associated with traditional osteoarthritis (OA) medications, a variety of new OA treatments are currently under investigation. These new agents can be categorized into several groups, including cartilage regeneration inducers, osteogenesis inhibitors, matrix degradation inhibitors, apoptosis inhibitors, and anti-inflammatory cytokines [10]. Potential therapeutic options include bone morphogenetic protein-7 (BMP-7), interleukin-1β (IL-1β), beta nerve growth factor, platelet-rich plasma (PRP), human serum albumin, etc. [10]. For example, bone morphogenetic protein-7 (BMP-7) is a biologic agent approved by the FDA for the treatment of impaired bone healing and spinal fusion [27]. A phase I study evaluating the safety and tolerability of BMP-7 in patients with symptomatic knee osteoarthritis reported promising results. Participants receiving doses of 0.1 mg and 0.3 mg of BMP-7 demonstrated greater improvements in symptoms and higher response rates [28]. A study involving 160 patients administered intra-articular injections of an IL-1β receptor antagonist. Although anakinra demonstrated good tolerability, no significant clinical improvements were reported compared to the placebo group [29]. Tanezumab is a monoclonal antibody targeting beta nerve growth factor that has been clinically evaluated for OA. A proof-of-concept study involving 450 patients with knee OA demonstrated that Tanezumab significantly reduced pain during walking. However, 68% of patients receiving Tanezumab experienced adverse events, prompting the FDA to request a suspension of the trials [30]. PRP contains various growth factors, such as transforming growth factor-beta 1 (TGF-β1), platelet-derived growth factor, vascular endothelial growth factor, insulin-like growth factor-1, and hepatocyte growth factor [31]. In a study by Wang-Saegusa et al., 312 knee OA patients received three intra-articular injections of autologous PRP. Six months later, significant improvements were observed in assessment metrics, including the VAS, SF-36, WOMAC, and the Lequesne index [32]. In 2014, a multicenter, randomized, double-blind study assessed the efficacy and safety of the Low Molecular Weight Fraction of 5% human serum albumin (LMWF-5A) in 329 knee osteoarthritis patients. At 12 weeks, the LMWF-5A significantly reduced pain compared to the vehicle control (−0.93 vs. −0.72; estimated difference: −0.25, *p* = 0.004), with no observed injection volume effect (*p* = 0.64). Adverse events were mild and similar in both the vehicle control group (47%) and the LMWF-5A group (41%) [33].

SVF can be differentiated from other cell types, such as chondrocytes, bones, and muscles [12]. The primary advantages of using SVF for OA treatment are largely attributed to the potential of the cells and their associated secretomes to mitigate degenerative processes [34]. While normal joint fluid contains mesenchymal stem cells (MSCs), their quantities are limited. Furthermore, although these cells can differentiate into new chondrocytes, the resulting cartilage is often fragile and highly susceptible to damage [35]. These limitations have prompted many researchers to explore the utilization of SVF as a means to address these shortcomings [34]. SVF exhibits significant potential in promoting neovascularization and angiogenesis [34]. A 2009 in vitro study indicated that endothelial progenitor cells (EPCs) and adipose-derived stem cells (ADSCs) can work synergistically to enhance neovascularization compared to their individual administration [36]. Moreover, SVF administration effectively reduces the overproduction of inflammatory cytokines and growth factors, such as TNF-α, IL-6, IL-12, and interferon-γ, in various disease models [37]. An in vitro study compared the effects of SVF and ASC on chondrocytes. Co-culturing with SVF resulted in greater cartilage matrix deposition and a higher proportion of chondrocytes than with ASC. In nude mice, SVF and chondrocyte mixtures outperformed ASC mixtures in cartilage matrix formation. Overall, SVF proved more effective for cartilage repair without prior cell expansion, indicating its potential in repair strategies [38]. In 2021, a network meta-analysis of seventy-nine randomized controlled trials (RCTs) involving 8761 patients assessed various intra-articular injectables, including ACS, bone marrow aspirate concentrate (BMAC), botulinum toxin, corticosteroids (CS), hyaluronic acid (HA), mesenchymal stem cells (MSC), ozone, saline placebo, PRP, plasma rich in growth factors (PRGF), and SVF. The results indicated that SVF had the highest P-scores for the VAS scores (P-score range = 0.8631–0.9927) and for the WOMAC at 12 months (P-score = 0.9044) [39].

Our study revealed that SVF effectively reduced pain and improved function in patients with OA, according to the results of these questionnaire scales. In particular, the WOMAC scores showed significant improvements between pre-intervention, 3, and 6 months after the intervention. Similarly, the VAS and KOOS scores significantly improved between pre-intervention and 6 months post-intervention. A previous network meta-analysis, which included 79 randomized controlled trials and 8761 patients with OA, corroborates these findings. Compared to the intra-articular saline injection group, the SVF group exhibited a mean VAS difference of −35.68 (95% confidence interval: −73.65 to 2.29) at 3 months and −38.98 (95% confidence interval: −54.65 to −23.31) 6 months after the intervention. Compared to the intra-articular saline injection group, the mean difference in WOMAC in the SVF group was 0.2 (95% confidence interval: −17.71 to 18.11) at 3 months post-intervention. Furthermore, 6 months after the intervention, the mean difference in VAS in the SVF group was −15.40 (95% confidence interval: −34.82 to 4.02) [39]. In 2020, a multicenter, prospective, double-blind, randomized placebo-controlled clinical trial recruited 39 adult patients with symptomatic knee OA. The patients were randomly assigned to one of three groups: high-dose SVF group (3.0 × 10^7^ SVF cells), low-dose SVF group (1.5 × 10^7^ SVF cells), or placebo group. After six months post-injection, the median percentage changes in WOMAC scores were 83.9% for the high-dose group, 51.5% for the low-dose group, and 25.0% for the placebo group. The changes in WOMAC scores for both the high-dose and low-dose groups were statistically significant compared to the placebo group (high-dose, *p* = 0.04; low-dose, *p* = 0.02), indicating a dose-dependent response to the treatment [11]. In terms of KOOS, another trial involving 123 patients with OA who were administered SVF injection reports that the KOOS improved significantly from a pre-intervention score of 51.4 ± 16.5 to 87.6 ± 7.7 (*p* < 0.05) 6 months after the intervention [40]. However, the long-term efficacy of SVF in OA requires further investigation. The WOMAC scores decreased to 16.33 ± 13.04 3 months after intervention and slightly increased to 20.22 ± 19.01 6 months post-intervention, suggesting that the treatment effect cannot be sustained for a long time. In contrast, the VAS and KOOS scores at 6 months after the intervention were greater than those at 3 months. These findings indicate that SVF treatment in patients with OA may have prolonged effects over time. A previous network meta-analysis of 79 randomized controlled trials involving 8761 patients with OA reports similar results. Compared to the intra-articular saline injection group, the mean difference in VAS in the SVF group was −38.98 (95% confidence interval: −54.65 to −23.31) at 6 months after the intervention. Compared to the intra-articular saline injection group, the mean difference in VAS in the SVF group was −30.38 (95% confidence interval: −54.77 to −5.99) at 12 months after the intervention. At 6 months post-intervention, the mean difference in VAS in the SVF group was −15.40 (95% confidence interval: −34.82 to 4.02), compared to the intra-articular saline injection group. The WOMAC in the SVF group showed a mean difference of −24.40 (95% confidence interval: −47.17 to −1.63) 12 months after the intervention, compared to the intra-articular saline injection group [39].

While no significant inter-observer agreement was found between the three observers, we discovered that the T2 values of the medial femorotibial joint were significantly higher than those of the lateral femorotibial joint (*p* = 0.0002), and the T2 values of the femoral condyles were significantly higher than those of the tibial plateau (*p* < 0.0001). These findings are consistent with the observations of Friedrich et al. [41].

The accuracy of T2 mapping for articular cartilage depends on both the imaging plane used and the observer’s experience. MRI is a highly sensitive, specific, and accurate noninvasive diagnostic modality for the detection of chondral defects [42] in the patellofemoral compartment of the knee, using arthroscopy as the reference gold standard. Harris, J.D. et al. demonstrated [43] large variations in the overall interobserver agreement for different regions of native cartilage. Furthermore, the magic angle effect is considered a key diagnostic challenge in the T2 mapping of articular cartilage. Mosher et al. [44,45] showed that the most significant changes in T2 values occur in the superficial cartilage layers due to the orientation of the collagen fibrils. This effect is more pronounced in these areas because the collagen fibrils are aligned more horizontally near the surface [46]. Another factor that affects T2 values in cartilage tissue is the tissue’s irregular and asymmetric shape when degenerated. This irregularity makes it difficult to create an accurate ROI for the entire cartilage, as defects and uneven surfaces result in partial volume effects and alter fiber orientation. Three-dimensional MRI now matches the diagnostic performance of two-dimensional MRI, with further improvements seen using 3.0-T field strength, 3D fast-spin-echo sequences, and multiplanar reformation [42].

Limited studies have discussed the effect of SVF on cartilage in patients with OA using MRI. In 2020, an RCT with 39 patients with symptomatic knee OA demonstrated that SVF had a dose-dependent positive effect on WOMAC but did not result in significant cartilage changes [11]. However, another RCT involving 95 patients with knee OA reports that SVF could repair cartilage defects, especially in the medial femoral and medial tibial condyle, as observed using the three-dimensional fat-suppressed spoiled gradient recalled echo sequence of MRI [47]. Our study revealed that SVF could relieve OA symptoms, corresponding to the findings of a previous study [11]. However, the effect of SVF on the changes in the component and volume of the cartilage remains unknown. The knee may undergo a degenerative process during the tracking period, resulting in cartilage-thinning changes. The observed decrease in cartilage thickness in the middle segment of the lateral femur following SVF treatment may be attributed to normal degeneration. The differences between the studies may stem from the variations in the participants and study designs. In our study, the results of the functional questionnaires indicated the potential efficacy of SVF in patients with OA. However, the observed improvement in clinical outcomes does not appear to have a direct correlation with the enhancement of cartilage quality. Further research with larger-scale comparative studies is necessary to validate the therapeutic effects of SVF and its impact on cartilage repair.

Our study has some limitations. First, we had a low number of participants due to funding constraints. Additionally, the follow-up loss rate was minimal. Further, large-scale studies are needed to validate our findings. Second, our study was a single-armed trial. To investigate the efficacy of SVF in treating OA, further cohort studies or RCTs are warranted. Third, we could only draw ROI manually with isotropic two-dimensional T2 mapping, potentially including the cortex and marginal spur within ROI. Adopting a higher-resolution isotropic three-dimensional T2 mapping technique could facilitate multiplanar reformatting. In addition, our study cohort consisted exclusively of female participants. This may limit the generalizability of our findings when applied to broader populations. Furthermore, comparing results between healthy volunteers and patients with knee OA is also essential.

## 5. Conclusions

Our study revealed that significant differences in the change in WOMAC (20.78 ± 7.03, *p* = 0.0183), VAS (2.00 ± 2.29, *p* = 0.0307), and KOOS (−11.01 ± 13.93, *p* = 0.0453) occurred between pre-intervention and 6 months post-intervention. In addition, SVF had a positive effect on the anterior superficial layer of the medial femoral cartilage and middle superficial layer of the lateral femoral cartilage, while the opposite effect was observed in the posterior superficial layer of the medial tibial cartilage. The decreased cartilage thickness observed in the middle segment of the lateral femur may be attributed to the degenerative process observed during the tracking period. No significant differences were observed in T2 values across most of the regions of cartilage in the tibia, femur, and patella. Furthermore, no definite correlation was found between relative changes in T2 values and WOMAC score, VAS, and KOOS at 3 or 6 months. However, further cohort studies or RCTs are necessary to validate our findings.

## Figures and Tables

**Table 1:** MRI parameters on a 3-T MRI scanner (Ingenia, Philips).

Sequence	T2 Map	T2WI mFFE	T1WI Coronal	PDFS Axial
Repetition time (ms)	2000	20	663	4925
Echo times (ms)	13, 26, 39, 52, 65, 78	5	20	30
Field of view (mm)	160 × 160	140 × 140 × 100	140 × 140 × 100	170 × 170
Slice thickness (mm)	3	1	2.5	2.5
Gap (mm)	0.3	−0.5	0	0
Flip angle (deg)	90	45	90	90
Matrix	296 × 236	176 × 174	280 × 231 × 40	339 × 270

**Table 2:** Patient age distribution (*n* = 9).

Variables	All (n = 9)	
	*n*	%
**Gender**		
Female	9	100.00%
**Age (Median ± IQR)**	59 ± 7	
**Age group (years)**		
50–54	2	22.22%
55–59	3	33.33%
60–64	3	33.33%
65–69	1	11.11%
**Height (Median ± IQR)** **(cm)**	163 ± 4	
**Weight (Median ± IQR)** **(kg)**	68 ± 12	
**BMI (Median ± IQR)** **(kg/m^2^)**	25.6 ± 5.0	
**Grade of osteoarthritis–right foot**		
2	5	55.55%
3	3	33.33%
Undergone knee replacement	1	11.11%
**Grade of osteoarthritis–left foot**		
2	7	77.77%
3	2	22.22%
**Underlying diseases**		
**Hypertension (HTN)**	4	44.44%
**Diabetes mellitus (DM)**	1	11.11%
**Heart failure**	1	11.11%
**Total live cells (Median ± IQR)**	2,810,000 ± 1,140,000	

**Table 3:** Questionnaire scores at different time points (*n* = 9).

Variables	Pre-Intervention		3 Months After the Intervention		6 Months After the Intervention	
	Means	SD	Means	SD	Means	SD
WOMAC score	41.00	25.67	16.33	13.04	20.22	19.01
VAS pain score	5.00	2.96	3.89	3.76	3.00	3.50
KOOS score	52.01	14.67	61.96	13.41	63.02	16.85

**Table 4:** Corresponding questionnaire scores at different time points (*n* = 9).

Variables	Pre-Intervention—3 Months After the Intervention			Pre-Intervention—6 Months After the Intervention		
	Mean Difference	SD Difference	*p* Value	Mean Difference	SD Difference	*p* Value
WOMAC score	24.67	25.00	0.0181 *	20.78	7.03	0.0183 *
VAS pain score	1.11	2.57	0.2310	2.00	2.29	0.0307 *
KOOS score	−9.95	13.38	0.0563	−11.01	13.93	0.0453 *

* Shows significance in *p* value.

**Table 5:** Baseline T2 values for cartilage (median ± interquartile range, IQR) (n = 9).

	Medial ^#^	Lateral ^#^	Medial & Lateral
Femur *	51.1 ± 14.3	47.9 ± 13.1	50.0 ± 12.3
Tibia *	40.8 ± 9.6	30.9 ± 4.5	35.1 ± 9.9
Femur & Tibia	45.3 ± 10.7	37.6 ± 17.0	42.3 ± 14.9

* Femur versus tibia, *p* < 0.0001. ^#^ Medial versus lateral, *p* = 0.0002.

**Table 6:** Baseline T2 values for cartilage (median ± interquartile range, IQR).

**Femur**	**Medial ^#^**	**Lateral ^#^**	**Medial & Lateral**
Deep *	44.9 ± 7.5	40.5 ± 8.4	42.9 ± 8.2
Superficial *	60.3 ± 21.1	50.1 ± 16.8	54.7 ± 18.6
Whole	51.1 ± 14.3	47.9 ± 13.1	50.0 ± 12.3
**Tibia**	**Medial ^#^**	**Lateral ^#^**	**Medial & Lateral**
Deep *	34.6 ± 7.2	28.5 ± 5.5	30.1 ± 6.6
Superficial *	45.0 ± 9.7	33.7 ± 6.7	39.3 ± 11.7
Whole	40.8 ± 9.6	30.9 ± 4.5	35.1 ± 9.9
**Patella**			
Deep *		40.1 ± 6.4	
Superficial *		53.1 ± 14.7	
Whole		45.9 ± 10.1	

* Deep versus superficial, *p* < 0.0001. ^#^ Medial versus lateral, *p* < 0.0001.

**Table 7:** T2 values in all segments (median ± interquartile range, milliseconds) (*n* = 9).

	Segments	Baseline	After SVF	Relative Changes	*p* Value
Medial Femur	Anterior deep	42.7 ± 8.2	42.3 ± 10.2	1.2 ± 6.6	0.9799
	Middle deep	42.6 ± 12.7	44.7 ± 10.7	0.3 ± 10.7	0.8603
	Posterior deep	47.4 ± 7.1	47.7 ± 10.2	0.0 ± 4.9	0.9399
	Anterior superficial *	55.3 ± 10.8	51.7 ± 5.7	6.7 ± 11.2	0.0052 *
	Middle superficial	63.0 ± 28.2	55.9 ± 31.9	5.2 ± 18.7	0.1167
	Posterior superficial	49.2 ± 37.8	50.1 ± 37.1	−7.0 ± 10.9	0.3225
	Whole	51.1 ± 14.3	48.2 ± 14.8	0.8 ± 7.9	0.6322
Medial Tibia	Anterior deep	35.5 ± 9.9	34.0 ± 7.5	0.2 ± 6.6	0.9399
	Middle deep	32.1 ± 6.5	29.5 ± 8.1	1.5 ± 7.2	0.2114
	Posterior deep	36.0 ± 8.7	34.4 ± 11.0	0.4 ± 8.4	0.8209
	Anterior superficial	41.5 ± 6.8	42.3 ± 17.8	−1.2 ± 12.2	0.3484
	Middle superficial	44.9 ± 20.0	44.0 ± 15.6	0.9 ± 10.9	0.6685
	Posterior superficial *	44.3 ± 4.1	47.4 ± 5.5	−2.8 ± 3.5	0.0027 *
	Whole	40.8 ± 9.6	39.3 ± 4.4	−1.0 ± 8.8	0.8603
Lateral Femur	Anterior deep	37.6 ± 12.4	39.8 ± 11.5	0.7 ± 8.3	0.6685
	Middle deep	39.2 ± 11.0	41.1 ± 7.1	0.2 ± 8.9	0.4637
	Posterior deep	46.8 ± 10.2	45.9 ± 9.1	1.1 ± 8.5	0.8999
	Anterior superficial	48.7 ± 24.4	45.8 ± 11.2	3.6 ± 14.6	0.1046
	Middle superficial *	53.1 ± 13.5	48.1 ± 14.3	3.7 ± 4.4	0.0131 *
	Posterior superficial	48.3 ± 12.8	46.0 ± 11.6	0.7 ± 7.9	0.7436
	Whole	47.9 ± 13.1	44.2 ± 9.8	1.5 ± 6.4	0.0954
Lateral Tibia	Anterior deep	26.2 ± 6.2	25.6 ± 4.1	0.0 ± 3.8	0.9399
	Middle deep	24.3 ± 3.7	23.8 ± 6.1	−1.4 ± 4.5	0.4954
	Posterior deep	31.2 ± 8.8	30.4 ± 6.8	−0.9 ± 6.4	0.6685
	Anterior superficial	35.9 ± 5.0	33.9 ± 8.0	1.3 ± 11.0	0.7057
	Middle superficial	31.3 ± 3.7	30.4 ± 6.3	−0.6 ± 3.7	0.1928
	Posterior superficial	37.4 ± 9.0	40.0 ± 9.5	−2.9 ± 7.4	0.1439
	Whole	30.9 ± 4.5	32.1 ± 5.0	−0.9 ± 3.2	0.2312
Patella	Deep	40.1 ± 6.4	39.8 ± 8.9	1.8 ± 6.8	0.2522
	Superficial	53.1 ± 14.7	49.7 ± 11.8	3.6 ± 10.2	0.2247
	Whole	45.9 ± 10.1	44.4 ± 6.6	2.2 ± 7.3	0.0959

* Shows significance in *p* value.

**Table 8:** Spearman’s rank correlation coefficients between questionnaires and T2 values for cartilage (*n* = 9).

	Relative Changes in T2 Values				
Relative changes in questionnaires		Subregion	Anterior superficial layer of medial femoral cartilage	Middle superficial layer of lateral femoral cartilage	Posterior superficial layer of medial tibial cartilage
	Questionnaires				
	WOMAC difference at 3 months	Spearman’s rho	−0.05913	−0.01626	0.36512
		*p* value	0.8278	0.9523	0.1644
	WOMAC difference at 6 months	Spearman’s rho	0.11234	0.03104	0.30599
		*p* value	0.6787	0.9091	0.2491
	VAS difference at 3 months	Spearman’s rho	0.2528	0.1763	0.10558
		*p* value	0.3448	0.5137	0.6972
	VAS difference at 6 months	Spearman’s rho	0.18594	0.03149	0.36889
		*p* value	0.4905	0.9078	0.1597
	KOOS difference at 3 months	Spearman’s rho	−0.2779	−0.29712	−0.15521
		*p* value	0.2973	0.2638	0.566
	KOOS difference at 6 months	Spearman’s rho	−0.4372	−0.38515	−0.1383
		*p* value	0.0904	0.1407	0.6095

**Table 9:** Cartilage thickness (median ± interquartile range, mm) (n = 9).

	Segments	Baseline	After SVF	Relative Changes	*p* Value
Medial Femur	Anterior	1.4 ± 0.5	1.3 ± 0.4	0.0 ± 0.5	0.8603
	Middle	1.2 ± 0.4	1.1 ± 0.4	0.0 ± 0.2	0.4037
	Posterior	1.0 ± 0.4	1.2 ± 0.5	−0.1 ± 0.3	0.2979
Medial Tibia	Anterior	1.1 ± 0.3	1.0 ± 0.1	0.0 ± 0.5	0.2522
	Middle	1.4 ± 0.3	1.5 ± 0.5	−0.1 ± 0.4	0.0739
	Posterior	1.5 ± 0.5	1.5 ± 0.6	−0.1 ± 0.3	0.3484
Lateral Femur	Anterior	0.9 ± 0.4	0.9 ± 0.5	0.0 ± 0.1	0.2979
	Middle *	1.3 ± 0.4	1.2 ± 0.3	0.1 ± 0.2	0.0131 *
	Posterior	1.1 ± 0.3	1.2 ± 0.3	0.0 ± 0.2	0.8603
Lateral Tibia	Anterior	1.5 ± 0.5	1.3 ± 0.5	0.0 ± 0.3	0.3824
	Middle	2.4 ± 1.0	2.2 ± 1.1	0.1 ± 0.4	0.3484
	Posterior	2.0 ± 1.0	1.8 ± 0.8	0.1 ± 0.4	0.4637
Patella	Whole	2.0 ± 1.1	2.1 ± 1.0	0.1 ± 0.3	0.2633

* Shows significance in *p* value.

## Data Availability

The authors declare that the data supporting the findings of this study are available within the paper.
